# La maladie de Forestier: une cause rare de dysphagie à ne pas méconnaître

**DOI:** 10.11604/pamj.2014.18.140.4710

**Published:** 2014-06-17

**Authors:** Wassia Kessomtini, Wafa Chebbi

**Affiliations:** 1 Unité de Médecine Physique, CHU Taher Sfar Mahdia, 5100 Mahdia, Tunisie; 2 Service de Médecine Interne, CHU Taher Sfar Mahdia, 5100 Mahdia, Tunisie

**Keywords:** Maladie de Forestier, dysphagie, Forestier disease, dysphagia

## Image en medicine

La maladie de Forestier ou hyperostose ankylosante sénile rachidienne est une maladie rhumatologique d’étiologie inconnue décrite pour la première fois en 1950 par Forestier and Rotes-Querol. Elle est caractérisée par une ossification des ligaments paravertébraux et des enthèses périphériques. Le plus souvent asymptomatique, la maladie de Forestier est de découverte fortuite sur des radiographies standards. Plus rarement, elle se manifeste par des douleurs cervicales, une raideur rachidienne ou une dysphagie. Nous rapportons l'observation d'une patiente âgée de 58 ans, aux antécédents de diabète 2, qui consultait pour une dysphagie haute aux solides évoluant depuis 6 mois, associée à des cervicalgies. La dysphagie s'améliorait par la flexion du rachis cervical et s'aggravait par l'extension. Il n'y avait pas de dyspnée, ni de dysphonie, ni d'altération de l’état général, ni notion de fausses routes. La mobilité rachidienne cervicale était conservée. Les examens ORL et neurologique étaient sans particularités. Le bilan biologique ne montrait pas de troubles métaboliques ni de syndrome inflammatoire. Les radiographies standards du rachis cervical révélaient des ostéophytes cervicaux antérieurs en regard de C4-C5 et C5-C6. Le transit oesogastroduodénal objectivait une compression de la paroi postérieure de l'oesophage en regard des ostéophytes. Le traitement était symptomatique reposant sur des anti-inflammatoires non stéroïdiens, des antalgiques et une rééducation adaptée.

**Figure 1:**
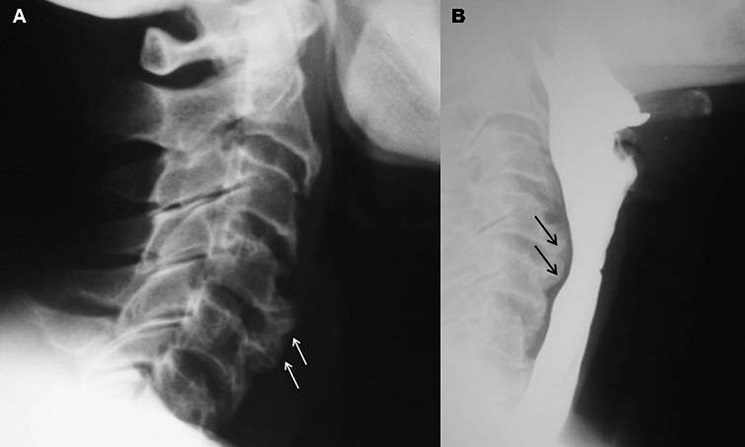
A) Radiographie du rachis cervical de profil: ostéophytes cervicaux antérieurs C4-C5 et C5-C6 (flèches blanches); B) Transit oesogastroduodénal: compression de la paroi postérieure de l'œsophage en regard des ostéophytes (flèches noires).

